# Identification of early biomarkers in a rabbit model of primary Candida pneumonia

**DOI:** 10.1186/s12879-019-4320-9

**Published:** 2019-08-06

**Authors:** Gang Lu, Chen Wang, Chunrong Wu, Lei Yan, Jianguo Tang

**Affiliations:** 1 Department of Trauma-Emergency & Critical Care Medicine, Shanghai Fifth People’s Hospital, Fudan University, No. 128 Ruili Road, Shanghai, 200240 China; 2 Department of Respiratory, Taizhou Municipal Hospital, No. 381 East Zhongshan Road, Taizhou, 318000 Zhejiang Province China

**Keywords:** sTREM-1, *Candida albicans*, Lung infection, Biomarkers, sTREM-1, SCD163

## Abstract

**Background:**

*Candida albicans* is an opportunistic pathogen, but since it also belongs to the normal fungal flora, positive sputum culture as the solely basis for the diagnosis of invasive *Candida albicans* pneumonia can easily lead to excessive antifungal therapy. Therefore, identification of a pneumonia biomarker might improve diagnostic accuracy.

**Methods:**

A rabbit model was established by inoculating 5 × 10^7^ cfu/mL *C. albicans* into the trachea of 20 rabbits with 20 rabbits as control group. Infection was monitored by chest thin-layer computed tomography (CT). 2 mL blood samples were collected daily during each infection and serum levels of potential biomarkers were measured by enzyme-linked immunosorbent assay (ELISA). Seven-day post-inoculation the rabbits were sacrificed by CO_2_ asphyxiation and lung tissue was histopathologically examined and blood was brought to culture. Data were statistically analyzed.

**Results:**

Infection became evident as early as day 3 post-inoculation. The levels of soluble triggering receptor expressed on myeloid cells-1 (sTREM-1), soluble hemoglobin-haptoglobin scavenger receptor (sCD163), procalcitonin (PCT) and tumor necrosis factor-α (TNF-α) were elevated in the experimental group compared to the control (*P* < 0.01), whereas the levels of C-reactive protein (CRP), interleukin-6 (IL-6), IL-8 and IL-10 showed no significant differences (*P* > 0.05). The dynamic curves of the levels of CRP, IL-6, IL-8, IL-10, SCD163 and TNF-α in both groups demonstrated a similar trend during infection but differences between the groups was observed only in the sTREM-1 levels. Receiver-operating characteristics (ROC) curve analysis showed that the sensitivity and specificity were 85 and 80% for sTREM-1 (cut-off value: 45.88 pg/mL) and 80 and 75% for SCD163 (cut-off value: 16.44 U/mL), respectively. The values of the area under the ROC curve (AUC_ROC_) of sTREM-1 and SCD163 were 0.882 (95% CI: 0.922–0.976) and 0.814 (95% CI: 0.678–0.950), respectively. Other markers did not exhibit significant differences.

**Conclusion:**

sTREM-1 and SCD163 might be suitable biomarkers for pneumonia.

## Background

A serious risk factor for patients in hospital intensive care units (ICUs) is the potential of contracting opportunistic infections from a variety of pathogens. The EPIC II study, which investigated the prevalence of infections in 1265 ICUs across 75 countries, showed that 51% of 13,796 cases contracted infections, of which 64% involved the respiratory system and 19% of them were caused by fungi [1].

Nevertheless, diagnosis of *Candida* pneumonia is a challenge, since *Candida* spp. are part of the normal flora and asymptomatic colonizers of various mucosal surfaces including the upper respiratory tract. There is a need to differentiate between primary isolated *Candida* lung invasion causing pneumonia and secondary hematogenously disseminated *Candida* reaching the lungs from elsewhere. Pneumonia caused by fungi is provisionally diagnosed by a combination of clinical and radiological findings, together with cultures and serology [2].

To detect the presence of infection and inflammation in patients, a wide variety of potential serum markers were investigated including factors involved in infection and inflammatory responses i.e., soluble triggering receptor expressed on myeloid cells (sTREM-1, a member of the immunoglobulin superfamily) [3–7], soluble hemoglobin-haptoglobin scavenger receptor (sCD163) [8–10], procalcitonin (PCT) [11], C-reactive protein (CRP) [12] as well as interleukin (IL)-6, IL-8, IL-10 and tumor necrosis factor alpha (TNF-α) [13–16]. However, the majority of studies that investigated biomarkers for infectious diseases focused on a relatively heavy load of septic infection and did not differentiate fungal from bacterial infections and to date only a few studies have focused on *C. albicans* infection [17]. Therefore, identification of marker for *Candida-*induced pneumonia (CP) would be highly desirable to facilitate the early diagnosis and effective treatment of patients. In the present study, we established a rabbit CP model and evaluated potential biomarkers (vide supra) using biochemical and statistical analyses for them to be useful for the early diagnosis and prognosis of CP.

## Methods

### Animals and microorganisms

All procedures involving animals were performed in accordance with the ethical standards of the participating institution and the Guidelines for the Humane Treatment of Laboratory Animals (Ministry of Science and Technology of the People’s Republic of China, Policy No. 2006 398), and were approved by the Institutional Animal Care and Use Committee of Shanghai Fifth People’s Hospital.

A total of 40, pathogen free, New Zealand white rabbits (males, 2 months old, 2.0–2.5 kg) were purchased from the Animal Center of the Shanghai Jiaotong University. The animals were reared in well ventilated, stainless steel, 60 × 80 cm rabbit cages (2 rabbits per cage) placed in a temperature-controlled room (20 m^2^) at 20–30 °C and 40–70% humidity. Lighting included a combination of natural light and fluorescence. Tap water and mixed pellet feed were provided daily. Rabbit cages and drinking bottles were sterilized using 0.1% benzalkonium bromide and bedding was changed every 2 days. The animals were observed for 5 days and used for experimentation in the absence of anomalous behavior.

**Fig. 1:**
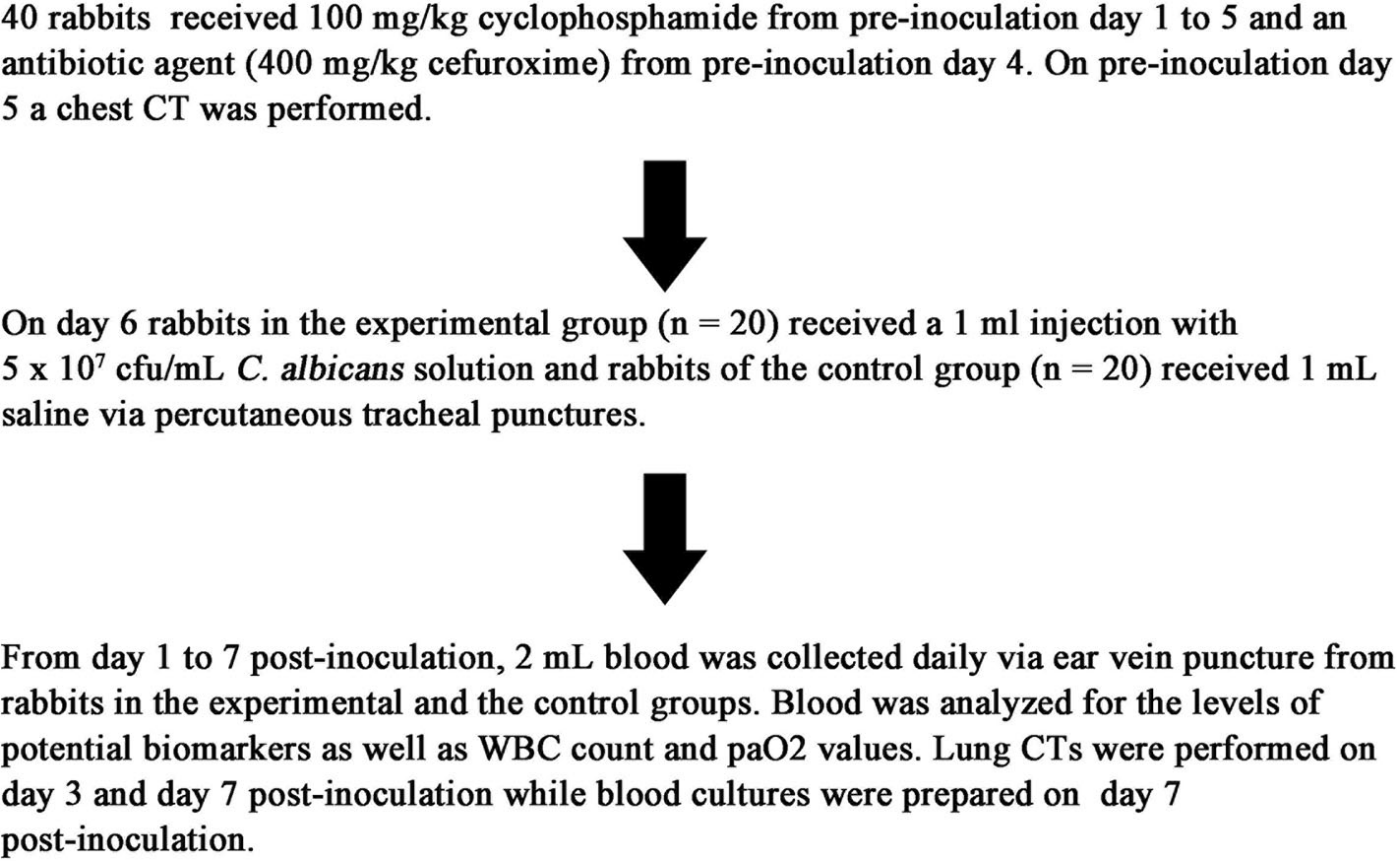
Establishment procedure of the *C. albicans* pneumonia rabbit model

All animals received 100 mg/kg cyclophosphamide via an ear vein injection daily from day 1 to 6 pre-inoculation to maintain a low immune status [18]. Bacterial infection was prevented with 400 mg/kg cefuroxime from day 4 onwards (Fig. 1). A standard strain of *C. albicans* (ATCC10235) was used for infection. Figure 1 describes the establishment procedure of the rabbit model.

### Establishment of a primary rabbit CP model

On day 6 pre-inoculation, rabbits were randomly divided into 2 groups of 20. The experimental group received a 1 mL injection of 5 × 10^7^ cfu/mL *C. albicans* solution via percutaneous tracheal puncture, while the control group received normal saline via the same route. The rabbits were anesthetized with 2 mL/kg intravenous injection of 3% sodium pentobarbital and carefully positioned on the surgical operating table. After trimming neck hair and disinfection with iodine, a towel with a hole was placed over the rabbit neck. Using sterile gloves, the position of the trachea was determined, and the trachea was fixed and punctured at a 45° oblique angle. After inoculation with 1.0 mL *C. albicans* solution, the rabbits were immediately moved to a dorsal elevated position and gently shaken for 5 min to allow the inoculum to enter the lower respiratory tract. Finally, the animals were returned to their respective cages to wake up naturally.

### Chest computed tomography imaging

Rabbits in the experimental and control groups were placed in the prone position after intravenous injection of 3% pentobarbital sodium (2 mL/kg) for chest thin-layer CT on day 1 pre-inoculation and days 3 and 7 post-inoculation (GE light speed 64VCT, USA). Two independent experts evaluated the CT images.

### Sample collection and measurements

From day 1 to 7 post-inoculation, 2 mL blood samples were collected daily from ear vein punctures of rabbits in the experimental and control groups. The blood gas parameter (PaO_2_) was measured with a gas analysis instrument (GEM Premier 3000, USA), and also white blood cell (WBC) counts in serum. The protein levels of TNF-α, sCD163, sTREM-1, PCT, CRP, IL-6, IL-8 and IL-10 in serum were measured using ELISA (Guangzhou Jianlun Biological Technology Co., Ltd.). The ELISA kits used were sTREM-1 (96 T kit, JL003211), PCT (JL002421), IL-6 (JL002052), IL-8 (JL001248), IL-10 (JL001132) and TNF-α (JL004161). ELSA analysis was performed according to the manufacturer instructions.

### Pathological analysis of rabbit lung tissue

On day 7 post-inoculation, the rabbits were euthanized with carbon dioxide (CO_2_) in a CO_2_ chamber and lung tissue biopsy was performed while blood samples were cultured to detect whether *C. albicans* was present. The neck muscles were blunt dissected and the trachea dissociated to separate lung from thorax on a sterile console. Lung tissue was visually observed for pathological changes, followed by collection of pathological specimens of lung tissue with the dimension 2.0 × 2.0 × 0.2 to 0.3 cm from each pulmonary lobe near the airways and specimens from the surrounding lung tissue area with visually obvious lesions or palpable nodules. Tissue samples were fixed by immersion in a 4-fold volume of 10% formalin and processed for periodic acid Schiff (PAS) and hematoxylin and eosin (HE) staining.

### Detection of pulmonary infection and CP diagnosis with thin layer CT scans

Rabbit lungs were examined for *C. albicans* infection using prone position thin CT scans after 3 and 7 days of inoculation. CP diagnostic imaging was graded as mild, moderate or severe based on comparing the lung CT images before inoculation and 7 days later. The actual lesion area covering the entire lobe or integration of multiple nodules close to or > 1 lobe was defined as severe; actual lesion area or the area after integration of multiple nodules < 1; more than 1/2 of the lung lobe involved was defined as moderate; nodules or actual lesion area < 1/2 of the lung lobe, or small patches shadow, circumscribed bronchopneumonia shadow was defined as mild. Finally, 7 rabbits were evaluated as mild CP, 6 rabbits were in the moderate CP group and 7 rabbits were in the severe group. Finally CP was also diagnosed based on the presence of *C. albicans* forms and pseudohyphae/hyphae in lung tissue visualized by PAS staining.

### Statistical analysis

SPSS ver. 16.0 software (SPSS, Chicago, Illinois, USA) was used to perform all statistical analyses. The WBC count and oxygenation index with normal distributions are reported as the mean ± SD. A Mann-Whitney U test was used to compare non-normally distributed data such as PCT, SCD163, sTREM-1, CRP, IL-6, IL-8, IL-10, and TNF-α levels and the results are expressed as median values (interquartile range). Normally distributed data from appropriate groups were compared using a *t*-test. Qualitative data are presented as proportions. Comparisons between the 2 groups were made using a chi-squared test. The Spearman correlation coefficient was used to determine the coefficient for each biomarker investigated. The values of the area under the ROC (receiver-operating characteristics) curves (AUC_ROC_) were used to establish the specificity and sensitivity of each biomarker. All *P*-values were 2-sided and *P*-values < 0.05 were considered to be statistically significant.

## Results

### Establishment of a rabbit primary CP model

To establish a rabbit primary CP model, animals were immunosuppressed with cyclophosphamide and bacterial infection was prevented by the administration of cefuroxime. *C. albicans* was inoculated into rabbits in the experimental group and the resulting infection was monitored by blood culture, lung biopsy and chest CT. Blood cultures from rabbits in the experimental (*n* = 20) and the control (n = 20) groups were both negative for *C. albicans.* However, *C. albicans* was detected on histopathology and cultures from lungs of rabbits in the experimental but not in the control group. No pathogenic bacteria were detected in all cultures.

**Fig. 2:**
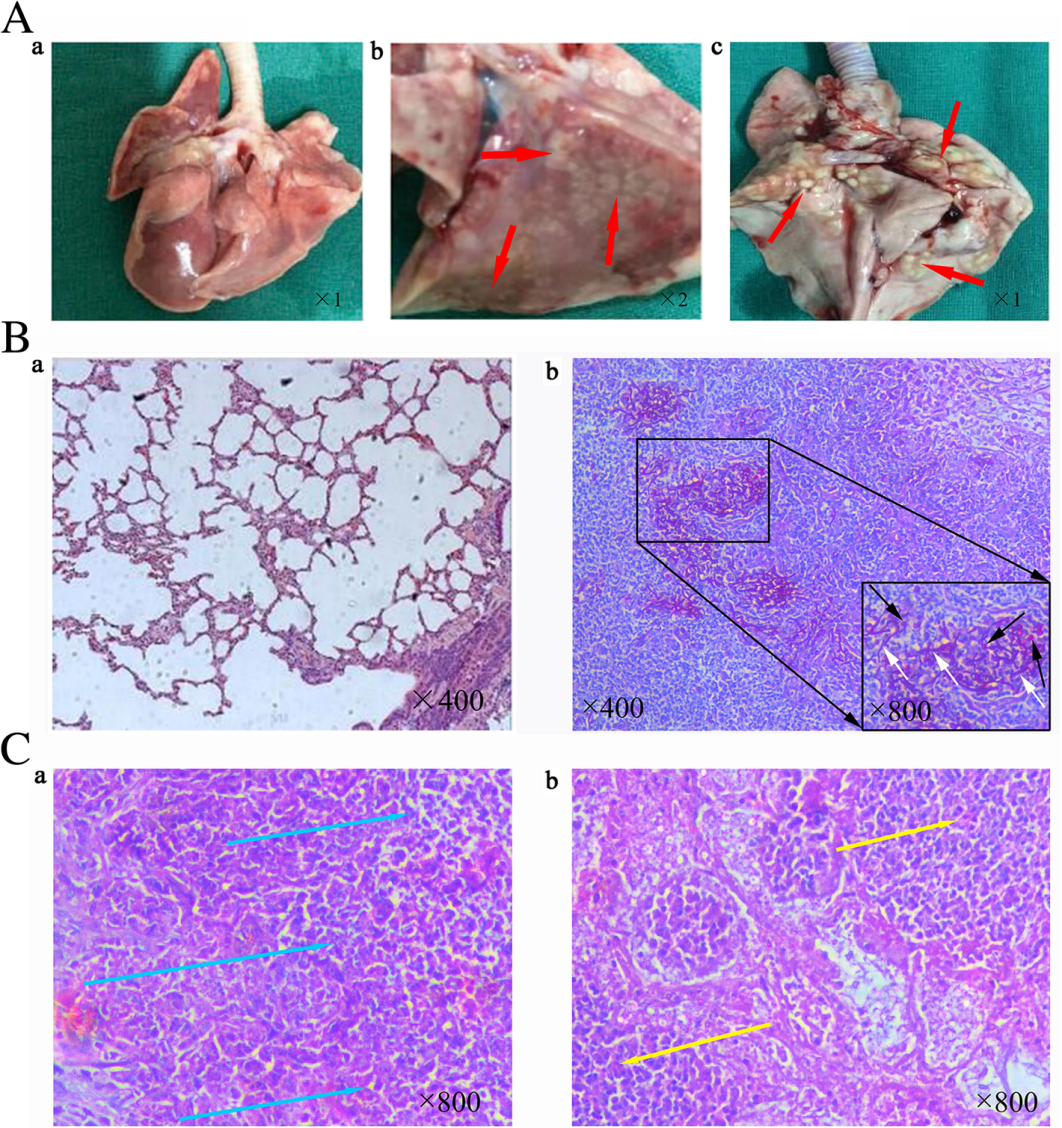
Macroscopic and histopathology of rabbit lung specimens. On the 7th day after *C. albicans* infection, lung specimens were visually examined. **a**) Histopathological images of (**a**) a normal healthy lung specimen from the control group (magnification × 1), (**b**) a lung from the *C. albicans* infection model group with nodular lesions (red arrows) (magnification × 2), (**c**) a lung from the rabbit *C. albicans* infection model group with diffuse nodular lesions (red arrows) (magnification × 1). B) PAS staining of (**a**) a normal healthy lung tissue and (**b**) upper panel, a lung tissue of a rabbit infected with *C. albicans* (magnification × 400), (**b**) lower panel, white arrows indicate pseudohyphae/hyphae, black arrows indicate yeast forms (magnification × 800). **c**) HE staining of (**a**) infiltration of surrounding lymphocytes (blue arrows) and (**b**) infiltration of surrounding eosinophilic granulocyte (yellow arrows) (magnification × 800)

On day 7 after *C. albicans* infection, lung histopathologic analysis was performed. In lungs isolated from the infected group, white diffuse nodular lesions were visually observed (Fig. 2a, b and c), while lungs from the control group showed no sign of infection (Fig. 2Aa). PAS staining of the infected lung tissue specimens showed pseudohyphae/hyphae appearing as thin and straight structures (white arrows) and round and oval yeast forms dyed purple and brown (black arrows) (Fig. 2Bb). H&E staining showed infiltrating lymphocytes (blue arrows) (Fig. 2Ca) and infiltration of eosinophilic granulocytes (yellow arrows) (Fig. 2Cb).

**Fig. 3:**
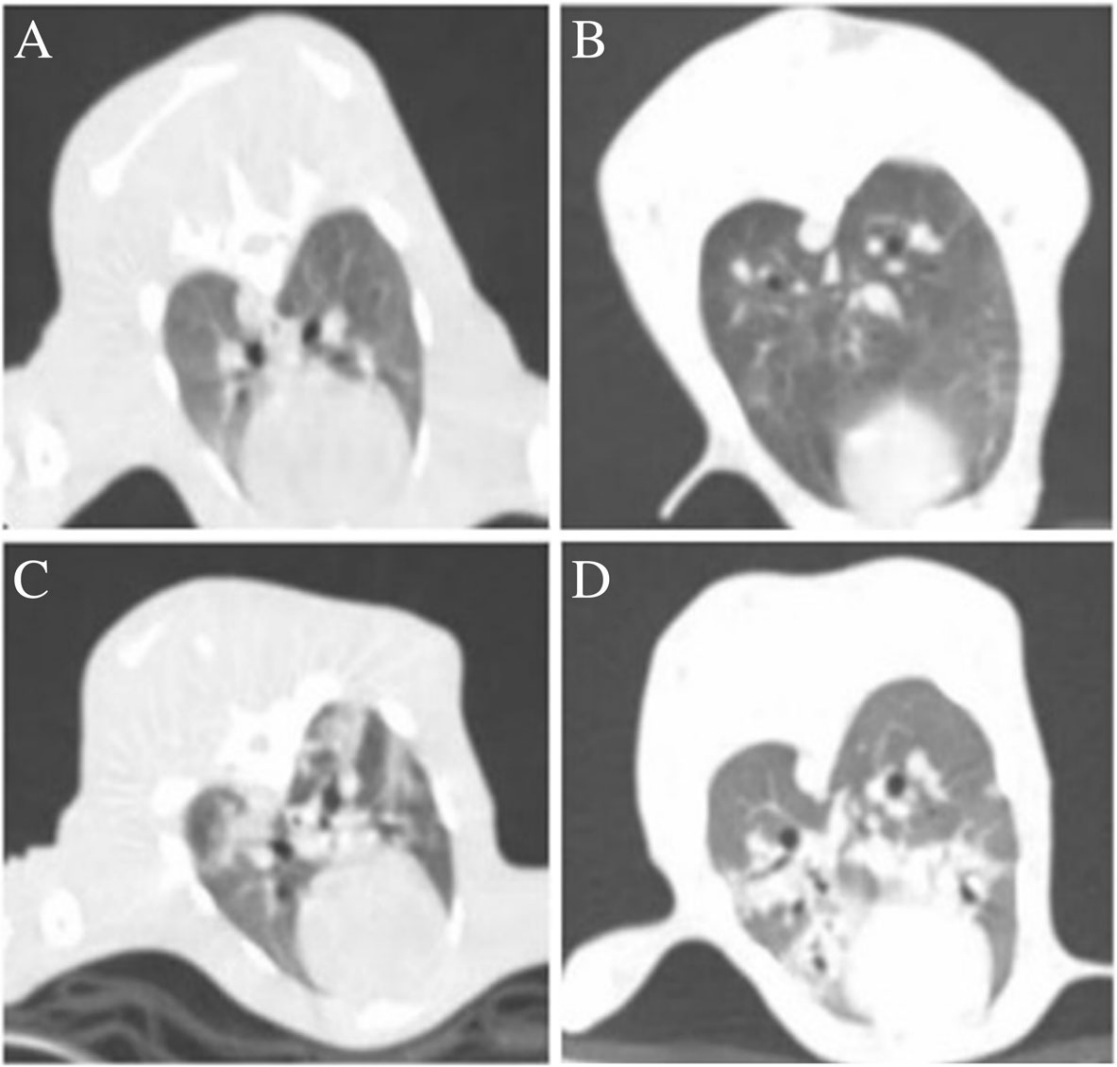
Chest CT images of a normal rabbit and a rabbit with severe white *Candida* pneumonia. (**a** and **b**) normal rabbit, (**c**) Day 3 post-inoculation demonstrating patchy shadows, substantial lesions and nodules, (**d**) Day 7 post-inoculation demonstrating larger lesions in the entire lung as compared to day 3 post-inoculation

On days 3 and 7 post-inoculation, chest thin-layer CT demonstrated progressive or stable lung lesions in all animals in the experimental group (Fig. 3c and d), while infection was absence in the control group (Fig. 3a and b). These observations showed that the rabbit CP model had been successfully established.

### Correlation analysis between biomarkers and the severity levels of CP

**Fig. 4:**
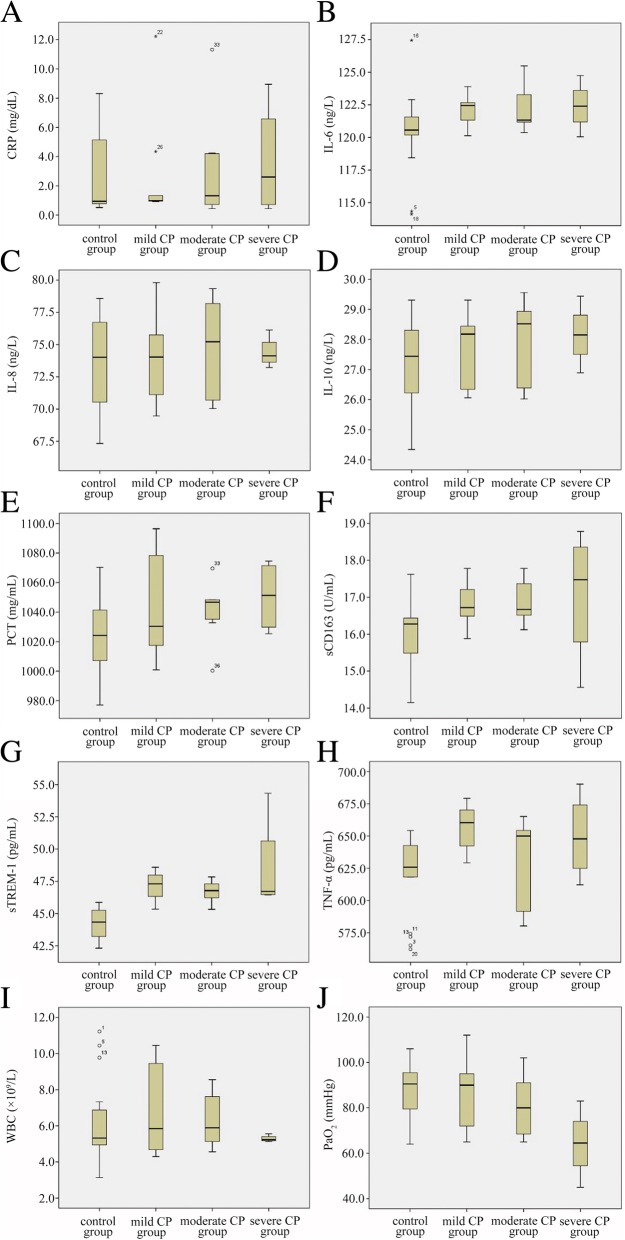
Correlation analysis between biomarkers and severity levels of CP (**a**) CRP; (**b**) IL-6; (**c**) IL-8; (**d**) IL-10, (**e**) PCT; (**f**) sCD163; (**g**) sTREM-1; (**h**) TNF-α; (**i**) WBC; (**j**) PaO_2_

During the first week after inoculation with *C. albicans*, blood samples were collected from rabbits in the experimental and control groups. Since lung CT scans showed signs of pulmonary infection as early as day 3 post-inoculation, we analyzed blood samples collected on day 3 to evaluate the levels of several potential biomarkers, as well as WBC counts. To assess respiratory function, PaO_2_ levels were measured. The results showed no significant difference in CRP levels between the control and mild experimental groups. However, as the disease worsened, the CRP level in the experimental group increased significantly and was significantly higher in the severe group than that in mild group (*P* < 0.01). The difference in IL-6 values between the control and experimental groups was significant, but this difference did not reflect the severity of pneumonia. There was no significant difference in IL-8 levels between the control and experimental groups. The IL-8 level did not increase with the severity of pneumonia. There was a significant difference in IL-10 levels between the control and experimental groups, but these did not reflect the severity of pneumonia. There was a significant difference in PCT levels between the control and experimental groups, when the PCT level increased with increasing severity of pneumonia, with a significantly higher level in the severe group compared with the mild group (*P* < 0.01). SCD163 levels were significantly higher in the experimental group than in control (*P* < 0.01). The values of sTREM-1 and TNF-α were higher in the experimental group than in the control group, but they did not increase with the severity of pneumonia. The WBC count was higher in the mild group than in control, but lower in the severe group compared with the control group. Thus, the WBC count did not reflect the infection and its severity. The PaO_2_ level was significantly lower in the experimental group than in the control group, which gradually decreased along with aggravation of pneumonia (Fig. 4).

### Dynamic curves of the serum levels of individual markers during infection

**Fig. 5:**
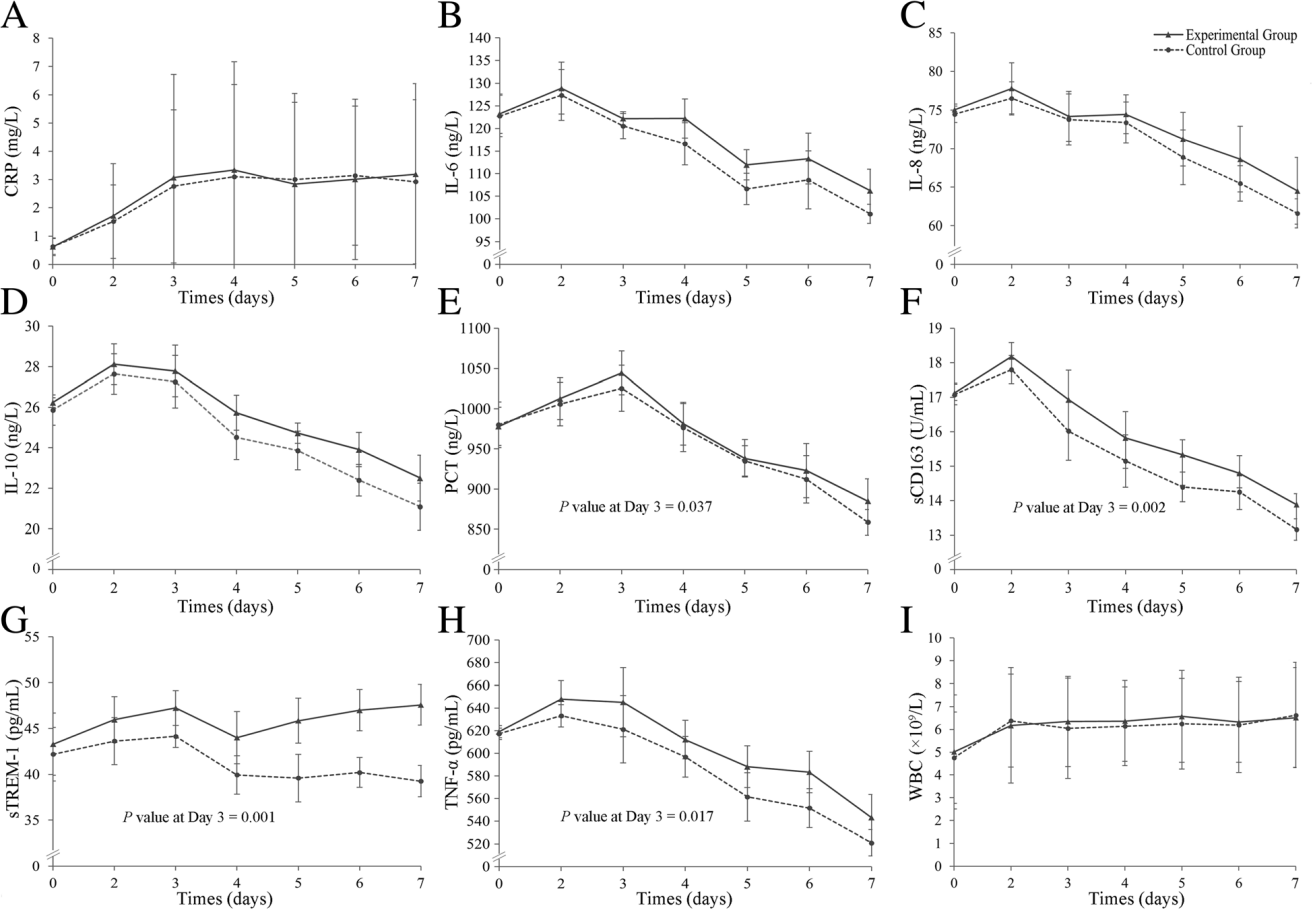
Dynamic curves of serum biomarker levels during *C. albicans* infection. The levels of the biomarkers were determined by ELISA analysis and plotted against the indicated times (days). The WBC count was quantified by routine blood examinations. (**a**) CRP, (**b**) IL-6, (**c**) IL-8, (**d**) IL-10, (**e**) PCT, (**f**) sCD163, (**g**) sTREM-1, (H) TNF-α and (**i**) WBC

To investigate further these markers, their serum levels were plotted against the pre- and post-inoculation times (days 2, 3, 4, 5, 6 and 7) to create dynamic curves. The curves of the levels of TNF-α, CRP, IL-6, IL-8 and IL-10, PCT, SCD163, as well as WBC counts, showed a similar trend in the experimental and control groups (Fig. 5). However, despite a similarity of the trend of the curve, significant differences between the experimental and the control groups in the levels of sCD163 (*P* = 0.002), TNF-α (*P* = 0.017), and sTREM-1 (*P* = 0.001), in addition to PCT (*P* = 0.037) were measured as early as day 3 post-inoculation. However, the interquartile ranges were very large for PCT, TNF- α and sCD163.

A significantly different trend was found in the sTREM-1 levels, with the levels in both groups gradually increasing immediately after inoculation and then declining until day 4. However, the levels in the experimental group started to increase thereafter, while the levels in the control group remained constant (Fig. 5), indicating a high potential of sTREM-1 as a biomarker.

### ROC curve analyses of biomarkers

**Fig. 6:**
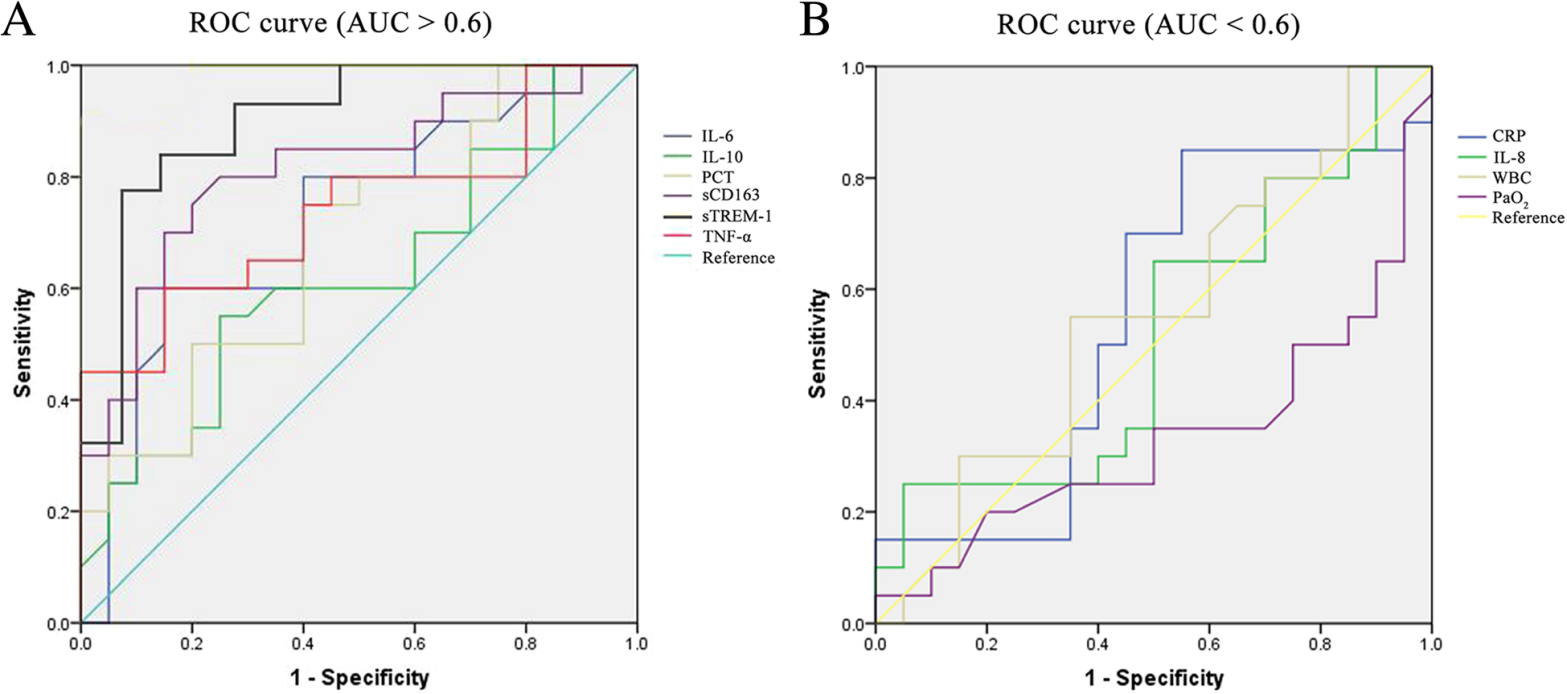
ROC curves of indicated potential CP biomarkers. (**a**) Biomarkers with AUC > 0.6 and (**b**) biomarkers with AUC < 0.6

To determine the specificity and sensitivity of biomarkers, ROC curve analyses were carried out (Table 1 and Fig. 6). When the cut-off value was set as 45.88 pg/mL, the I^2^ for the sensitivity and specificity of sTREM-1 for CP diagnosis was 85 and 80%, respectively, and the value of the AUC_ROC_ was 0.882 (95% CI: 0.922–0.976). I^2^ values for the sensitivity and specificity of sCD163 (the cut-off value 16.44 U/mL) were 80 and 75%, respectively, and AUC_ROC_ was 0.814 (95% CI: 0.678–0.950). As shown in Table 1, sTREM-1 and SCD163 demonstrated higher sensitivity and specificity as well as reliability compared to other candidate markers such as PCT, CRP, TNF-α and IL-6, IL-8 and IL-10. Especially, sTREM-1 showed the highest specificity and sensitivity as a biomarker for CP and its AUC_ROC_ value was the closest to 1.

**Table 1:** ROC curve analysis for potential biomarkers for CP diagnosis

	AUC (95% CI)	Cut-off value	Sensitivity	Specificity
PCT	0.685 (0.420, 0.850)	1027.84 ng/L	75%	65%
sCD163	0.814 (0.678, 0.950)	16.44 U/mL	80%	75%
sTREM-1	0.882 (0.922, 0.976)	45.88 pg/mL	85%	80%
CRP	0.55 (0.363, 0.737)	0.944 mg/L	70%	55%
IL-6	0.724 (0.563, 0.844)	121.26 ng/L	70%	60%
IL-8	0.514 (0.333, 0.702)	73.63 ng/L	60%	50%
IL-10	0.620 (0.444, 0.796)	27.50 ng/L	60%	55%
TNF-α	0.74 (0.583, 0.897)	638.89 pg/mL	70%	60%
WBC	0.553 (0.371, 0.734)	5.38 × 10^9^/L	55%	55%,
PaO_2_	0.333 (0.159, 0.506)	78 mmHg	50%	25%

PCT: procalcitonin, sCD163: soluble hemoglobin-haptoglobin scavenger receptor, sTREM-1: soluble triggering receptor expressed on myeloid cells-1, CRP: C-reactive protein, IL-6: interleukin-6, IL-8: interleukin-8, IL-10: interleukin-10, TNF-α: tumor necrosis factor-α, WBC: white blood cell, PaO_2_: blood gas parameter, AUC: value of area under the ROC curve

## Discussion

In the present study a potential value of sTREM-1 and sCD163 has been demonstrated in a rabbit model of Candida pneumonia. It has been reported that sTREM-1 levels in the bronchoalveolar lavage fluid of patients with bacterial or fungal pneumonia is significantly higher compared to patients with viral or atypical pneumonia, and it has been suggested that sTREM-1 could be used as a potential marker to differentially diagnose pneumonia [19]. In addition, a meta-analysis by Shi et al. suggested that sTREM-1 is a useful biomarker in ICU patients suffering from bacterial lung infections, but that further studies are required to confirm the ideal cut-off value [20]. In an effort to identify specific and sensitive biomarkers for the early diagnosis and prognosis of CP, we first established a rabbit model for primary CP. Mice are frequently used as animal models to study microbial infection because of their cost, ease of handling, technical feasibility and availability of strains [21, 22]. In the present study, we chose rabbits in order to take advantage of larger animals, which allowed us to sample the blood repeatedly for analysis of serum biomarkers and to visualize anatomical details by CT during the progression of *C. albicans* infection [23]. Histopathological examinations revealed pseudohyphae/hyphae and yeast forms in the *C albicans* infected rabbit lung tissues similar to findings in humans [24, 25]. Blood samples on day 3 post-inoculation, when lung CT scans revealed evidence of pulmonary infection, revealed higher serum levels of PCT, sCD163, sTREM-1 and TNF-α in infected rabbits compared to the controls (*P* < 0.01), but no significant differences were found in CRP, IL-6, IL-8 and IL-10 levels or PaO_2_ between the groups.

To determine the specificity and sensitivity of markers, we performed ROC curve analyses and assessed the diagnostic values. Among the markers examined, sTREM-1 showed the highest sensitivity and specificity of 85 and 80% (cut-off value 45.88 pg/mL), respectively, and the AUC_ROC_ value was 0.882 (95% CI: 0.922–0.976) indicating diagnostic accuracy_._ Also sCD163 showed a relatively high sensitivity and specificity of 80 and 75% (cut-off value 16.44 U/mL), respectively, and the AUC_ROC_ value was 0.814 (95% CI: 0.678–0.950). Other markers did not demonstrate promising values in the ROC curve analyses.

The diagnostic values of sTREM-1 and sCD163 have previously been extensively studied, and despite some contradictory reports sTREM-1 was successfully used as a marker for fungal or bacterial infection of the lungs [7, 19, 20]. Several studies have reported that the level of sCD163 in plasma was positively correlated with the severity of sepsis and proposed that sCDl63 could serve as a sepsis biomarker [9, 10].

However, in the present study disturbances in the levels of the sepsis markers by bacterial co-infections cannot be excluded, since no bacterial infection control was included and most studies on the biomarkers were carried out in the presence of bacterial infections. Nevertheless, the preliminary results of the present study may justify prospective monitoring of a human patient population. Another limitation of the present study was the small sample size. Further large scale studies will be required to confirm our findings and conclusions.

Taken together with other studies that sTREM-1 and sCD163 were elevated in patients with bilateral lung infiltrates caused by bacterial or fungal pneumonia [19] and more generally in sepsis [10] and the elevation is consistent with an invasive disease either from pneumonia or other causes of sepsis which can be due to fungal pneumonia as demonstrated in this study or bacterial pneumonia as demonstrated in humans [7, 20], both factors might serve as marker for CP diagnosis.

## Conclusion

We anticipate that measurement of both sTREM-1 and sCD163 levels in serum might, beside their function as indicators for bacterial infections, serve for the improvement of early CP diagnosis.

## Data Availability

The datasets used and/or analyzed during the current study are available from the corresponding author on reasonable request.

## Abbreviations

CP: *Candida* caused pneumonia
CRP: C-reactive protein
ICUs: intensive care unit
IFDs: invasive fungal diseases
IL-10: interleukin-10
IL-6: interleukin-6
IL-8: interleukin-8
TNF-α: tumor necrosis factor-α
PaO_2_: blood gas parameter
PCT: procalcitonin
sCD163: soluble hemoglobin-haptoglobin scavenger receptor
SIRS: Systemic inflammatory response syndrome
sTREM-1: soluble triggering receptor expressed on myeloid cells-1
WBC: white blood cell
